# Perseverations and switching during a semantic verbal fluency task

**DOI:** 10.1002/alz.087944

**Published:** 2025-01-03

**Authors:** Neha Dubey, Jayanti Basu, Amitabha Ghosh

**Affiliations:** ^1^ Department of Applied Psychology, University of Calcutta, Kolkata, West Bengal India; ^2^ Apollo Multispeciality Hospital, Kolkata, West Bengal India

## Abstract

**Background:**

It is believed that delayed perseveration on a semantic verbal fluency (SVF) test may occur due to impaired working memory (WM). However, the relation between perseveration and switching, and the self‐generated interference one has to overcome when switching back to a previously visited subcategory has not been explored. In this study we wanted to see: 1) if perseverative errors in SVF test require switching and subsequent switch back to a previously explored subcategory; (2) if this trend would parallel cognitive decline.

**Method:**

We studied three groups, amnestic mild cognitive impairment (aMCI) (n = 30), Alzheimer’s disease (AD) (n = 30) and healthy controls (HC) (n = 60) based on their performance on an SVF task along with tests of executive function. Our primary analysis, focussed on the association of switching and switch back with the first perseverative error.

**Result:**

Perseveration was significantly associated with switch back for individuals with cognitive decline and healthy controls (χ^2^ = 24.88, p = .000359). Fewer switches and switchbacks were needed to trigger a perseveration as we moved along the continuum of cognitively healthy to cognitively impaired (HC>aMCI>AD). Finally, across all groups, perseverations were a result of more switchbacks (Wald chi‐square = 8.909, p = .003) rather than other factors.

**Conclusion:**

These findings suggest that it is switching and eventual switch back to a previous subcategory, and not the distance from the index word alone, that trigger perseverative errors in SVF tasks.

